# Cross-cultural validation of the Turkish Four-Dimensional Symptom Questionnaire (4DSQ) using differential item and test functioning (DIF and DTF) analysis

**DOI:** 10.1186/s12875-016-0449-4

**Published:** 2016-05-11

**Authors:** Berend Terluin, Pemra C. Unalan, Nurver Turfaner Sipahioğlu, Seda Arslan Özkul, Harm W. J. van Marwijk

**Affiliations:** Department of General Practice and Elderly Care Medicine, and the EMGO Institute for Health and Care Research, VU University Medical Center, Van der Boechorststraat 7, 1081 BT Amsterdam, The Netherlands; Department of Family Medicine, Marmara University Medical Faculty, Başıbüyük Mah. Maltepe Başıbüyük Yolu Sok. No:9/1. Maltepe, 34854 Istanbul, Turkey; Department of Family Medicine, Cerrahpasa Medical Faculty, Istanbul University, 34303 Cerrahpasa, Istanbul, Turkey; Primary Care Research Centre, Institute of Population Health, Williamson Building, Oxford Road, Manchester, M13 9PL UK

**Keywords:** Distress, Depression, Anxiety, Somatization, Cross-cultural validation, Differential item functioning, Cultural beliefs

## Abstract

**Background:**

The Four-Dimensional Symptom Questionnaire (4DSQ) is originally a Dutch 50 item questionnaire developed in primary care to assess distress, depression, anxiety and somatization. We aimed to develop and validate a Turkish translation of the 4DSQ.

**Methods:**

The questionnaire was translated using forward and backward translation, and pilot testing. Turkish 4DSQ-data were collected in 352 consecutive adult primary care patients. For comparison, gender and age matched Dutch reference data were drawn from a larger existing dataset. We used differential item and test functioning (DIF and DTF) analysis to validate the Turkish translation to the original Dutch questionnaire. Through additional inquiry we tried to obtain more insight in the background of DIF in some items.

**Results:**

Twenty-one items displayed DIF but this impacted only the distress and depression scores. Inquiry among Turkish people revealed that the reason for DTF in the distress scale was probably related to unfavourable socio-economic circumstances. On the other hand, the likely explanation for DTF in the depression scale appeared to be grounded in culturally and religiously determined optimistic beliefs. Raising the distress cut-offs by 2 points and lowering the depression cut-offs by 1 point ensures that individual Turkish 4DSQ scores be correctly interpreted.

**Conclusions:**

The Turkish translation of the 4DSQ (named: “Dört-Boyutlu Yakınma Listesi”, 4BYL) measures the same constructs as the original Dutch questionnaire. Turkish anxiety and somatization scores can be interpreted in the same way as Dutch scores. However, when interpreting Turkish distress and depression scores, DTF should be taken into account.

**Electronic supplementary material:**

The online version of this article (doi:10.1186/s12875-016-0449-4) contains supplementary material, which is available to authorized users.

## Background

The Four-Dimensional Symptom Questionnaire (4DSQ) is used to support the evaluation of patients with (suspected) mental health problems in primary care settings [1]. The 4DSQ is originally a Dutch questionnaire, developed in primary care to measure distress, depression, anxiety and somatization [2, 3]. The distress dimension covers the emotional consequences of stress and coping. A high score in this dimension, combined with low scores on depression, anxiety and somatization, is typical for normal responses to stress. The depression dimension taps on symptoms of moderate and severe depressive disorder, and reflects the probability of having a depressive disorder severe enough to warrant specific treatment [4]. The anxiety dimension encompasses the kind of symptoms that are characteristic of anxiety disorders, and the anxiety score reflects the probability of having one or more anxiety disorders severe enough to require specific treatment [5]. The somatization dimension covers the kind of physical symptoms that usually are manifestations of bodily distress [6].

We wished to make the 4DSQ available in the Turkish language for a number of reasons. First, unlike most other mental symptom questionnaires around the world (of which many are available in the Turkish language), the 4DSQ is specifically developed and validated in primary care [3]. Second, the 4DSQ includes a distress scale, next to depression and anxiety scales, thereby facilitating the distinction between “normal” responses to stress, loss and adversity (which are extremely prevalent in primary care) and “pathological” depressive and anxiety disorders. With the recent publication of the DSM-5 [7] the issue of distinguishing normal reactions and true disorders has gained in importance, especially regarding the diagnosis of major depressive disorder [8, 9]. Third, not only are there many Turkish speaking people in Turkey, but there are also large populations of Turkish immigrants all over Europe. Migration is a well-known risk factor for mental health problems [10]. Thus, a Turkish translation of the 4DSQ might not only be interesting for Turkish primary care providers but also for providers outside Turkey.

Therefore, we aimed to develop the Turkish 4DSQ and to validate it against the original Dutch questionnaire. This paper describes the procedure of translating the 4DSQ into the Turkish language (i.e., linguistic validation), and the subsequent assessment of measurement equivalence of that translation compared with the original Dutch 4DSQ (i.e., psychometric validation). We hypothesised that the Turkish 4DSQ was equivalent to the original Dutch questionnaire.

## Methods

### Translation

Largely in accordance with the recommendations of the MAPI Research Institute [11], we created a Turkish version of the 4DSQ that was as similar as possible to the original questionnaire. A Turkish family physician (PCU) built a team of two translators and three reviewers, and acted as process coordinator. The developer of the questionnaire (BT) was involved at an early stage. After a conceptual analysis, a forward translation was made by one of the translators, a native Turkish psychiatrist living and working in the Netherlands. A second forward translation, created by an unknown translator in the Netherlands, was already available. Both translations were reviewed by the reviewers, who were all Turkish family physicians. Differences between the translations were discussed with the translator and the developer. A consensus translation was then presented for back-translation to a second independent translator who was a Turkish medical secretary, born in the Netherland, who had lived there till the age of 13. The back-translation was then reviewed and compared with the original questionnaire by the consultant and developer. This lead to the establishment of a revised preliminary Turkish version, that was subsequently presented to the reviewers for clinical review. The reviewers independently identified several items requiring minor adjustments. The resulting version was then pre-tested by each reviewer/physician in at least ten primary care patients. Then the reviewers analysed the responses and identified two items requiring additional modifications. After this adjustment, the Turkish 4DSQ was finalized and named the “Dört-Boyutlu Yakınma Listesi” (4BYL).

### Measurement

The 4DSQ contains 50 items, measuring distress (16 items), depression (6 items), anxiety (12 items) and somatization (16 items) [3]. The 4DSQ asks how often during the past week respondents have experienced certain symptoms providing the opportunity to respond on a 5-point scale from “no” to “very often or constantly”. However, in order to eliminate exaggerating response tendencies, the responses are coded on a 3-point scale before calculating scale scores: “no” = 0, “sometimes” = 1, “regularly”/”often”/“very often or constantly” = 2. This way of scoring aims to provide more weight to the presence of symptoms than to their subjective severity. The 4DSQ was used as a pen-and-paper version.

### Participants

Turkish 4DSQ-data were collected in consecutive adult patients at their first visit to the Marmara University Family Medicine outpatient clinics in Istanbul, Turkey. Patients were personally approached in the waiting room and specifically instructed not to skip any questions. The Dutch reference 4DSQ-data were drawn from a large database of primary care patients with suspected mental health problems, who had completed the 4DSQ within the framework of routine care in a primary care health centre in Almere, the Netherlands. An age and gender matched sample of patients was randomly selected from this database.

### Ethical approval

The Turkish study protocol was approved by the Marmara University Medical Faculty Ethical Committee (Ref. 70737436–050.06.04). Written informed consent was obtained from all Turkish participants before issuing the questionnaire. No ethical approval was obtained for the Dutch part of the study because, according to Dutch regulations, no ethical approval is needed for the collection of patient data during routine care and the use of anonymized data collected this way.

### Analysis

#### Initial analyses

Missing item scores were imputed using the response function method [12]. Differences in mean 4DSQ scale scores were tested using t-tests. In addition, we calculated Cronbach’s alpha as a measure of internal consistency reliability and obtained 2000 bootstrap estimates of the difference between the groups using the ‘psych’ package [13] in R 3.1.2. [14].

#### Unidimensionality

We assessed unidimensionality by multi-group confirmatory factor analysis (CFA) using the ‘lavaan’ package in R [15]. We fitted one-factor models for each scale, allowing for correlations between residual variances of items sharing specific content. To account for the ordinal character of the item scores, the items were treated as ordered variables. Criteria for unidimensionality included a comparative fit index (CFI) and Tucker-Lewis index (TLI) >0.95 and a root mean square error of approximation (RMSEA) <0.06 [16].

#### Differential item functioning

We used differential item functioning (DIF) analysis to evaluate measurement equivalence, i.e., whether the Turkish translation measured the same constructs as the original Dutch 4DSQ (see Additional file 1 for an explanation of the DIF methodology). DIF analysis assumes that the responses to the items of a scale (e.g., a depression scale) reflect an underlying latent trait (e.g., depression). The method examines whether these item responses, in relation to the underlying latent trait, are the same in different groups [17–19]. When these responses can be demonstrated to be the same in Turkish and Dutch primary care patients (i.e., when the items ‘function’ the same way in both groups), it can be assumed that the scale measures the same construct in both groups and, consequently, that the Turkish 4DSQ has the same validity as the original Dutch questionnaire [3–5]. Moreover, in the presence of measurement equivalence, the Turkish scores can be interpreted in the same way as the Dutch scores. We chose two methodologically different methods [19, 20], the non-parametric Mantel-Haenszel (M-H) method [21] and the parametric hybrid ordinal logistic regression (HOLR) method [22].

The M-H-method uses the sum score of a scale’s items as ‘matching variable’ and calculates ‘standardized mean differences’ (SMDs). Conventionally, an SMD of 5 % of the item score range (in this case 0.1 points) indicates a clinically important degree of DIF [23]. We chose *p* <0.001 to account for multiple testing. Items with DIF were removed from the matching variable to ‘purify’ the matching variable. The M-H-method detects mainly ‘uniform’ DIF. We used the freeware program jMetrik 2.1 (www.itemanalysis.com) [24].

For the HOLR-analysis we used the package ‘lordif’ in R [22]. The HOLR-method combines item response theory (IRT) with ordinal logistic regression (OLR) [22]. OLR models the odds of endorsing each of the ordered response categories of an item as a function of one or more ‘determinants’, in this case the latent trait and group membership. IRT-analysis is used to calculate theta-scores, which are subsequently used as matching variable. When group membership as a determinant results in a substantial improvement of the prediction of the item responses, ‘uniform’ DIF is present. Inclusion of the interaction term between matching variable and group membership allows for the testing of ‘non-uniform’ DIF. We used a significant (*p* <0.001) increase in the model’s explained variance (McFadden’s R^2^) by 2 % or more as criterion for total DIF [22]. The HOLR-method ‘purifies’ the matching variable by estimating group-specific parameters for the DIF-laden item.

#### Differential test functioning

To evaluate the effect of item level DIF on the 4DSQ scale scores we compared the raw scale scores (i.e., the ordinary sum of the item scores) with estimates of the DIF-free scores across both groups. We used Rasch analysis, a one parameter IRT model, to obtain theta-scores [25]. We used the DIF-free items as anchor-items to estimate theta-scores in both groups on the same scale. The item parameters of the DIF-laden items were estimated separately for Turkish and Dutch patients. The raw (i.e., DIF-laden) scale scores by group were then plotted against the DIF-free theta-scores. The effect of item level DIF on the scale score (i.e., DTF) was evidenced by the distance between the group-specific curves. We used jMetrik 2.1 to perform the Rasch analyses.

#### Sample size

There are no established rules for the sample size requirements for the cross-cultural validation of questionnaires, but there are some recommendations for specific techniques. Scott et al. recommend 200–300 subjects per group for DIF-analysis [26], and Rouquette & Falissard recommend 300 participants for factor analysis [27]. Therefore, to stay on the safe side, we aimed to include 350 Turkish participants.

#### Additional inquiry

To obtain insight in the background of the DIF that was discovered, we presented the results to a convenience sample of Turkish speaking people in our personal networks, living in Turkey or in the Netherlands. They were asked to reflect on the meaning of the DIF-items in order to discern why these items were either more or less severe for Turkish people or seemed not to measure exactly the same constructs as the Dutch counterpart items did. Results were discussed in the research team.

## Results

### Initial analyses

A total of 352 Turkish patients (73 % female) agreed to participate. Their mean age was 37.4 (SD = 14.5). The matched Dutch sample consisted of 352 patients (73 % female) with a mean age of 38.3 (SD = 14.5). In the Dutch sample 145 item scores (0.82 %) were missing. In the Turkish sample only 2 item scores (0.0001 %) were missing. The missing item scores were successfully imputed. Table 1 presents mean 4DSQ scores and Cronbach’s alpha values.

**Table 1:** Cronbach’s alpha values, mean 4DSQ-scores and standard deviations of the study groups

		Cronbach’s alpha			Mean scores (SD)		
4DSQ-scales	scale range	Turkish	Dutch	p	Turkish	Dutch	p
Distress	0–32	0.90	0.93	0.002	12.6 (8.1)	19.9 (9.0)	0.000
Depression	0–12	0.86	0.92	0.001	3.0 (3.4)	4.4 (4.2)	0.000
Anxiety	0–24	0.84	0.92	0.000	5.2 (5.1)	7.2 (6.9)	0.000
Somatization	0–32	0.86	0.89	0.035	11.1 (7.0)	15.3 (8.2)	0.000

### Unidimensionality

Multi-group CFA confirmed one-factor models for the 4DSQ-scales across both groups (Table 2). The residual covariance of four item pairs and one item triplet needed to be freely estimated in order to obtain the desired model fit. The fit indices suggested adequate model fit.

**Table 2:** Results of the multi-group confirmative factor analyses (CFA)

4DSQ-scales	Chi-square	df	p	CFI	TLI	RMSEA	90 % CI
Distress^a^	442.41	204	0.000	0.994	0.993	0.058	0.050–0.065
Depression^b^	30.99	16	0.014	0.999	0.999	0.052	0.023–0.079
Anxiety	236.93	108	0.000	0.993	0.991	0.058	0.048–0.068
Somatization^c^	356.90	200	0.000	0.989	0.987	0.047	0.039–0.055

Chi-square = mean and variance adjusted model chi-square statistic

*CFI* comparative fit index

*TLI* Tucker-Lewis index

*RMSEA* root mean square error of approximation

90 % *CI* 90 % confidence interval of the RMSEA

^a^ residual correlations allowed between item pairs #20-#39 (sleep problems), and #47-#48 (upsetting events)

^b^ residual correlations allowed between item pairs #33-#46 (suicidal ideation)

^c^ residual correlations allowed between item pair #15-#16 (thoracic symptoms), and item triplet #9-#12-#13 (gastro-intestinal symptoms)

### DIF-analysis

The M-H-analysis identified DIF in 20 out of the 50 items, whereas the HOLR-method identified 12 items with DIF (both methods identified 21 items with DIF; Table 3). Ten items were more severe for Turkish patients while 11 items were less severe. Three items (#47, #48, #49) exhibited mixed uniform and non-uniform DIF.

**Table 3:** Items identified as having differential item functioning (DIF)

Scale/item #	English description	Turkish description	M-H-method		HOLR-method	
Distress			Direction	SMD	Direction	ΔR^2^
	*During the past week, did you suffer from:*	*Geçtiğimiz hafta aşağıdaki belirtilerden şikayetiniz oldu mu?*				
# 17	feeling down or depressed?	keyifsizlik/isteksizlik	**+**	0.40	**+**	3.87
# 19	worry?	birşeyleri kafaya takıp durmak	**-**	−0.16		
# 20	disturbed sleep?	huzursuz uyuma	**-**	−0.18		
# 22	lack of energy?	bitkinlik	**+**	0.23		
	*During the past week, did you feel:*	*Geçtiğimiz hafta aşağıdaki duyguları yaşadınız mı?*				
# 26	easily irritated?	çarçabuk asabileşmek	**-**	−0.32	**-**	5.36
	*During the past week, did you:*	*Geçtiğimiz hafta aşağıdaki durumları hissettiniz mi?*				
# 37	no longer feel like doing anything?	artık içinizden hiç bir şey yapmak gelmediğini/hiç bir şeyden zevk almadığınızı	**+**	0.19		
	*During the past week:*	*Geçen hafta:*				
# 41	did you easily become emotional?	Çabuk duygusallaştığınız oldu mu?	**+**	0.31	**+**	2.60
# 47	did you ever have fleeting images of any upsetting event(s) that you have experienced?	Birden, daha önce başınızdan geçmiş ağır bir olayın görüntüleri veya izleri zihninize (aklınıza) doluştu mu?	**+**	0.38	**+**	6.47
# 48	did you ever have to do your best to put aside thoughts about any upsetting event(s)?	Daha önce başınızdan geçmiş ağır bir olayı zihninizden uzaklaştırmak (aklınızdan çıkarmak) için olağanüstü bir çaba harcamak zorunda kaldınız mı?	**+**	0.26	**+**	3.45
Depression						
	*During the past week, did you feel:*	*Geçtiğimiz hafta aşağıdaki duyguları yaşadınız mı?*				
# 28	that everything is meaningless?	herşeyin manasız olduğunu	**-**	−0.15		
# 34	that you can’t enjoy anything anymore?	hiç bir şeyden zevk almadığınızı	**-**	−0.18		
# 35	that there is no escape from your situation?	hiç bir çıkış yolunuzun kalmadığını	**-**	−0.29	**-**	8.49
	*During the past week:*	*Geçen hafta:*				
# 46	did you ever think “I wish I was dead”?	Keşke ölsem dediğiniz oldu mu?			**+**	2.55
Anxiety						
	*During the past week, did you suffer from:*	*Geçtiğimiz hafta aşağıdaki belirtilerden şikayetiniz oldu mu?*				
# 21	a vague feeling of fear?	sebepsiz/yersiz korkular	**-**	−0.25		
# 23	trembling when with other people?	başkalarının yanında sıkılma/bunalma	**+**	0.44	**+**	10.46
	*During the past week, did you feel:*	*Geçtiğimiz hafta aşağıdaki duyguları yaşadınız mı?*				
# 27	frightened?	korku içinde olma	**-**	−0.28	**-**	3.58
	*During the past week:*	*Geçen hafta:*				
# 49	did you have to avoid certain places because they frightened you?	Korktuğunuz için belirli yerlerden geçmemek/oralarda bulunmamak için çaba harcadınız mı?	**+**	0.19	**+**	3.84
Somatization						
	*During the past week, did you suffer from:*	*Geçtiğimiz hafta aşağıdaki belirtilerden şikayetiniz oldu mu?*				
# 1	dizziness or feeling light-headed?	baş dönmesi veya kafanızda bir hafiflik hissi	**-**	−0.35	**-**	2.49
# 6	excessive sweating?	aşırı terleme	**-**	−0.17		
# 11	shortness of breath?	bunaltı	**+**	0.19		
# 14	tingling in the fingers?	parmaklarda karıncalanma	**+**	0.16		

Method: *H-M* Mantel-Haenszel, *HOLR* hybrid ordinal logistic regression

Direction: − = more severe for Turkish patients, **+** = less severe for Turkish patients

*SMD* standardized mean difference; *ΔR* ^*2*^ difference in R^2^ value (x100)

The distress item with the largest amount of total DIF (ΔR^2^ = 6.47 %), item #47 (“fleeting images of any upsetting event(s)”), was responsible for a mean increase in raw distress score of 0.38 scale points in Turkish patients, holding the true level of distress constant. The worst depression item (total DIF: ΔR^2^ = 8.49 %), item #35 (“feeling there is no escape from your situation”), was responsible for a mean decrease in raw depression score of 0.29 scale points in Turkish patients, holding the true level of depression constant.

### DTF-analysis

**Fig. 1:**
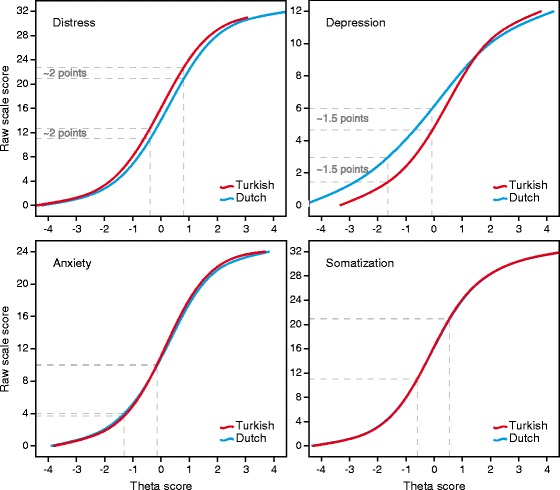
Differential test functioning (DTF) of the 4DSQ-scales. Raw 4DSQ scale scores as a function of the DIF-adjusted theta scores for distress, depression, anxiety and somatization, by language group (red curves: Turkish, blue curves: Dutch). Conventional Dutch cut-off points and corresponding Turkish cut-off points are indicated by dashed lines. The vertical distance between the Dutch and corresponding Turkish cut-off points indicate differential test functioning

Figure 1 shows the raw 4DSQ scale scores as functions of the theta scores, by group. Note that the theta scores produced by the Rasch analyses reflected the unbiased position of the patients on the latent traits underlying the 4DSQ-scales. The impact of item level DIF on the total scale score was apparent by the vertical distance between the curves for Turkish and Dutch patients.

With respect to the distress scale, we can see that the Turkish patients obtained a higher distress score (about 2 scale points on a scale range of 32 points) than the Dutch patients when they had the same true level of distress (represented by the theta score). This difference was about the same across the whole range of the distress scale except for the extremes. If left uncorrected, this DIF will result in some overrating of distress in Turkish patients.

Regarding the depression scale, there was a relatively large difference between the raw scale scores of Turkish and Dutch patients (about 1.5 points on a scale range of 12 points). Given a true level of depression represented by a theta score of 0 (this is the mean severity level of the symptoms), Turkish patients scored on average 1.5 points lower than Dutch patients (4.5 versus 6). If left uncorrected, this DIF will lead to underrating of depression and underdetection of depressive disorder in Turkish patients, compared to Dutch patients.

With respect to the anxiety and somatization scales, the item level DIF did not have any substantial impact on the raw scale scores.

### Background of DIF

Results were discussed with 9 persons living in Turkey and 20 native Turkish speaking immigrants living in the Netherlands for more than 20 years. The discussions focused on the items that were responsible for differential functioning of the distress and depression scales, i.e., the distress items that were less severe for Turkish patients and the depression items that were more severe for Turkish patients.

#### Distress

Low mood (item #17, “feeling down or depressed”) appeared to be difficult to translate into Turkish. Our translation of “keyifsizlik/isteksizlik”, literally meaning “malaise/reluctance”, turned out to be less severe for Turkish patients than the Dutch word “neerslachtigheid” is for Dutch patients. According to our informants the words “keyifsizlik/isteksizlik” represented emotions that could change within hours, defining mood states that were indeed less severe than what is denoted “depressed mood” in medical jargon. The other DIF-laden distress items (#22, #37, #41, #47, and #48) appeared to be correctly translated. Our informants suggested that the difficult socio-economic situation in Turkey, given the economic crisis, had made Turkish people more sensitive to specific features of distress as described by these items.

#### Depression

The most problematic depression item was item #35 (“feeling that there is no escape from your situation”). The translation appeared to be linguistically correct. However, our informants suggested that in the Turkish culture it is considered to be a shame to be that hopeless. One female informant said that “every bad thing has its worse”. This basic optimism appeared also to have a religious Islamic dimension, as observed by the difference in responses between more and less religiously engaged informants. A male informant said “As a religious person I cannot accept this statement! Because I believe in God, I know there is always a solution that He will show me.” A common Turkish expression is “When one door closes, God opens another door”. Religious beliefs also appeared to be at play in the response of Turkish people to item #28 (“feeling that everything is meaningless”). Our informants expressed a deeply felt conviction that “every creature in the world has an aim and its life has a meaning”. The feeling that everything is meaningless implies criticism towards the world’s creator, as the whole world is God’s work. The informants suggested that in item #34 (“can’t enjoy anything anymore”) probably the use of the word “zevk” (“pleasure”) was responsible for DIF because “zevk” possesses stronger physical connotations than “enjoy” (in Dutch: “genieten”). This would make it more difficult for Turkish people to admit any loss of pleasure.

## Discussion

The 4DSQ scales demonstrated good internal consistency reliability as evidenced by Cronbach’s alpha values well above 0.80. The Turkish values were somewhat lower than the Dutch values, probably due to the smaller variability of the Turkish scores.

We found that the Turkish translation of the 4DSQ contained 21 items that functioned differently from their original Dutch counterpart items. These Turkish items differed mainly in ‘severity’ (10 items were more severe and 11 items were less severe). Only three items also differed in ‘discrimination’. Therefore, we can conclude that the Turkish 4DSQ items and hence the Turkish 4DSQ scales measure the same constructs as the Dutch items and scales. The differential test functioning (DTF) analysis showed that the Turkish 4DSQ anxiety and somatization scales are equivalent to the corresponding Dutch scales. This is the case despite the existence of non-equivalence at the item level. The likely reason why item level DIF did not impact the scale score, is that the effects of items that were more severe for Turkish patients were counteracted by the effects of items that were less severe, causing DIF to cancel out on the scale level. Unfortunately, such cancelling out of DIF effects did not occur with the distress and depression scales. In case of the distress scale, most DIF-laden items (6 out of 9) were less severe for Turkish patients, causing them to score higher on the distress scale compared to their true level of distress (as estimated by the theta-score), and compared to Dutch patients.

The depression scale also suffered from imbalanced DIF. Three out of four DIF-laden items were more difficult for Turkish patients than for Dutch patients, causing lower raw depression scores in comparison to their true level of depression. The DTF analysis revealed that the effect of DIF on the scale level occurred only in the mild and moderate range of the depression trait. Turkish patients with a moderate degree of depression (corresponding to a theta-score of 0 or a raw depression score of 6 in Dutch patients) scored on average about 1.5 scale points less than Dutch patients with an equivalent true depression level. The reason was that for Turkish patients the thresholds to score on items #28, #34 and #35 were much higher than for Dutch patients. However, when the depression was really severe (corresponding to a theta-score of 1 or a raw depression score of 8–9 in Dutch patients) Turkish patients scored just like Dutch patients. Thus, because some depression items were more severe for Turkish patients than for Dutch patients, mild and moderate raw depression scores in Turkish patients did not have the exact meaning as in Dutch patients and tended to underestimate the true level of depression.

Whenever an item functions differently in two groups, there must be a reason for this that must be found in differences between the groups involved. Our primary interest concerned differences in language (translation) and culture. However, other differences between the groups, such as religion, marital status, educational level, or occupational status, might also be responsible. It should be noted that differences in gender and age have been controlled for by matching the Dutch sample on these characteristics. Other variables were not assessed.

The linguistic validation procedure, with forward and back translations and pilot reviews, provides some protection against flawed translations. Nevertheless, a translation might not catch the exact meaning and cultural loading of the original item. The translated item may still be a good indicator of the trait, but the item might be more or less severe than the original one. In many instances the exact meaning and nuance of a given word or expression in one language is difficult or sometimes even impossible to grasp in another language. This is true for depression-related words and expressions in the Turkish language [28]. This kind of DIF is not always a big problem on the level of the scale score provided that DIF-items that are more severe balance DIF-items that are less severe. This was the case with the Turkish 4DSQ anxiety and somatization scales.

A special situation occurs when a translated item, which may be perfectly translated from a linguistic point of view, acquires a different cultural loading. This was the case in 3 of the 6 depression items. Two items expressed severe pessimistic/desperate thoughts and feelings, unacceptable to Turkish people. Therefore the thresholds for experiencing and reporting such desperate thoughts and feelings were much higher for Turkish patients than for Dutch patients. In other words, whereas Dutch patients fall prey to pessimism and despair at relatively low levels of depression, Turkish patients need to be really severely depressed before they experience and report these kind of thoughts and feelings.

There is some supportive evidence in the literature. Religion may offer some protection against mental ill health through providing a secure attachment to God and meaning in life [29]. In a comparison of depressive symptom profiles between native Dutch people and Turkish-Dutch immigrants the item “feeling trapped” appeared to be much more severe for Turkish-Dutch people than for native Dutch [30]. “Feeling trapped” and “feeling there is no escape” probably refer to the same pessimistic, desperate mind set. In a study comparing depressive symptoms across British and Turkish psychiatric outpatient samples the same phenomenon was suggested as Turkish patients scored lower on pessimism [31].

We must acknowledge some limitations to this study. First, the Turkish and Dutch samples were not representative of all Turkish and Dutch speaking people as they were selected from specific urban regions. Second, female gender dominated both samples, reflecting the usual gender distribution in primary care patients in many countries. Because of the matching of the Dutch group (for gender and age) this female preponderance has not likely biased the DIF/DTF assessment. However, the relative underrepresentation of the male gender might have hidden any limited DIF/DTF that might have occurred only in men. The Turkish sample was not large enough to allow for the examination of gender-related DIF/DTF. Third, other sample characteristics than gender and age, such as education and physical health status, were unknown and could not be taken into account.

This paper offers an example of the validation of a questionnaire translation using DIF/DTF analysis, the most powerful method to establish whether a translated questionnaire measures the same constructs in the same way as the original questionnaire. The experience so far with other 4DSQ translations [32–34] is that usually some DIF/DTF is revealed in one or more scales. This DIF/DTF is, however, generally not severe enough to threaten the validity of the scales, and adjustment of cut-off points usually suffices to enable the correct interpretation of individual scores.

## Conclusions

The Turkish 4DSQ (4BYL) can be used to measure distress, depression, anxiety and somatization in primary care patients. The Turkish anxiety and somatization scales were found to be equivalent to the corresponding Dutch scales and, therefore, the scores can be interpreted in the same way as the Dutch scores. However, Turkish patients tend to score higher on the distress scale and lower on the depression scale compared to Dutch patients. In order to retain the same meaning of the cut-off points, those of the Turkish distress scale should be raised by 2 points. In addition, the cut-off points of the depression scale should be lowered by 1 point. In future research, other Turkish items could be tested to replace the worst DIF-items of the distress and depression scales. For the time being, adjustment of cut-off points for distress and depression seems to be the most practical solution.

## Availability of data

The dataset used cannot be made available due to the fact that it contains indirect identifiers (age and gender) which could compromise the participants’ anonymity.

## Additional file

Additional file 1: Explanation of differential item functioning (DIF) analysis. (PDF 267 kb)

## Abbreviations

4BYL: Dört-Boyutlu Yakınma Listesi (Turkish for: Four-Dimensional Symptom Questionnaire)
4DSQ: Four-Dimensional Symptom Questionnaire
CFA: Confirmatory factor analysis
CFI: Comparative fit index
DIF: Differential item functioning
DTF: Differential test functioning
HOLR: Hybrid ordinal logistic regression
M-H: Mantel-Haenszel
RMSEA: Root mean square error of approximation
SMD: Standardized mean differences
TLI: Tucker-Lewis index
