# Supplementary material for: Cross-cultural validation of the Turkish Four-Dimensional Symptom Questionnaire (4DSQ) using differential item and test functioning (DIF and DTF) analysis
Source: BMC Fam Pract. 2016 May 11;17:53. doi: 10.1186/s12875-016-0449-4 (PMC4864907; doi:10.1186/s12875-016-0449-4)
Supplement: Additional file 1: — Explanation of differential item functioning (DIF) analysis. (PDF 267 kb) [file 12875_2016_449_MOESM1_ESM.pdf]

Additional file 1

Differential item functioning

Differential item functioning (DIF) analysis assumes that the responses to the items of a scale (e.g., a depression scale) reflect an underlying latent trait (e.g., depression). The method examines whether these item responses, in relation to the underlying latent depression trait, are the same in different groups [1-3]. The relation between an item and the underlying latent trait, of which the responses to that item are assumed to be the expression, is characterized by the item parameters 'severity' and 'discrimination' and can be visualized by the item characteristics curve (ICC). Figure 1 shows an ICC of a dichotomous item (i.e., an item with two response options: yes/no). The curve displays the probability of a positive response as a function of the underlying trait. If this were the latent trait of depression, the probability of a positive response to this depression item increases with an increasing level of depression severity. The 'severity' parameter of the item is the level of the latent trait associated with a 50% probability of a positive response. It reflects the severity of the item in terms of the level of depression required to endorse the item.

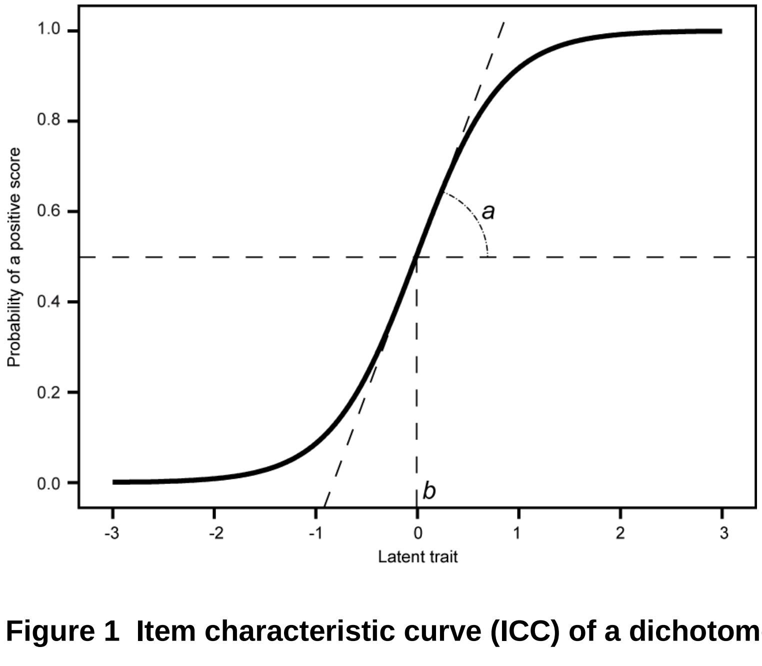

Figure 1 Item characteristic curve (ICC) of a dichotomous item
The curve displays the probability of a positive response to the item as a function of the underlying trait. The 'severity' of the item (*b*) is the level of the latent trait where the probability of a positive response is 50%. The 'discrimination' of the item is the slope of the curve (*a*). The latent trait scale is an arbitrary scale.

The ‘discrimination’ parameter of the item is represented by the slope of the curve. The steeper the curve, the better able the item is to distinguish people who are high on the latent trait (i.e., who are more severely depressed) from people who are low on the trait (i.e., who are less severely depressed). Because the ‘discrimination’ parameter also reflects the correlation between the item and the latent trait, this parameter indicates how well an item measures the latent trait. Different items have different ‘severity’ and ‘discrimination’ parameters in a given group of respondents.

Differential item functioning (DIF, i.e., when an item does not ‘function’ the same way in different groups) means that an item has different ‘severity’ or ‘discrimination’ parameters (or both) across different groups. An item can be more ‘severe’ for one group than for another group. This is called uniform DIF because the item is uniformly (i.e., over the whole range of the latent trait) more severe for one group than the other (Figure 2, left panel). In addition, an item can be more ‘discriminative’ in one group than another group. This is called non-uniform DIF because the item is relatively more severe for one group in one part of the latent trait scale, but relatively more severe for the other group in the other part of the scale (Figure 2, right panel). Non-uniform DIF suggests that the item does not measure the latent trait equally well in both groups. In the group in which the item demonstrates the lowest discrimination (i.e., in which the slope of the ICC is more gentle), the item either measures (partly) something different than the latent trait or the item score contains more measurement error (i.e., the item is less reliable). DIF-analysis aims to examine whether or not the severity and discrimination of an item is the same in different groups. If the items of a scale have the same severity and discrimination parameters in different groups, then it can be assumed that the scale measures the same construct in these groups. However, when a depression item is less severe in group A than in group B (Figure 2, left panel), people in group A tend to get higher depression scores than people in group B while having the same ‘true’ level of the latent depression trait. The presence of DIF prevents the meaningful comparison of depression scores across different groups. When a depression item is less discriminative in group A than in group B (Figure 2, right panel), because in group A the item is measuring something else or has more measurement error, people tend to get higher depression scores when their ‘true’ level of depression is relatively low. But, when the ‘true’ level of depression is relatively high, people in group A tend to get lower depression scores because the item does not measure depression as well as it does in group B.

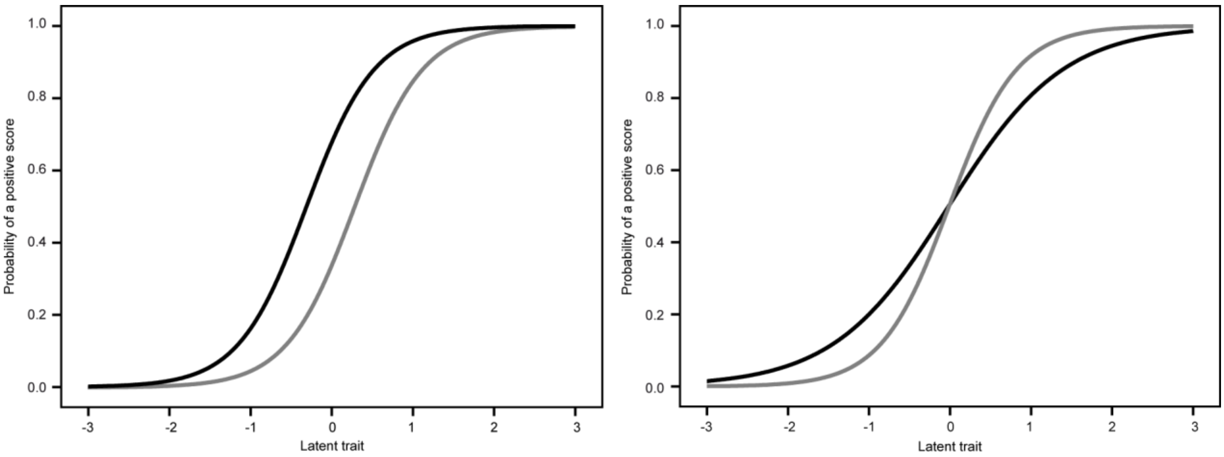

Figure 2 Examples of uniform DIF (left panel) and non-uniform DIF (right panel)
Both figures display the ICC's of one item in two groups in which the item differs in 'severity' (left panel) or in 'discrimination' (right panel). In uniform DIF (left panel) group A (black curve) has a uniformly higher (or equal) probability of a positive score than group B (grey curve). In non-uniform DIF (right panel) group A (black curve) has a higher probability of a positive score than group B (grey curve) in the lower part of the latent trait scale while, reversely, group B has a higher probability of a positive score than group A in the higher part of the scale.

In DIF-analysis the item parameters severity and discrimination are compared between two (or more) groups. All DIF-analysis methods need to match the groups according to the latent trait. Therefore, they need a (measurable) variable that approximates the (immeasurable) latent trait. This so-called 'matching variable' is usually somehow constructed based on the information of the item scores. However, when some of the items contain DIF, the matching variable will contain DIF and will not provide an unbiased approximation of the latent trait. Therefore, the matching variable needs to be 'purified', i.e., DIF needs to be removed from the matching variable. This is accomplished in different ways in different DIF-analysis methods.

Reference List

1. Zumbo BD: *A Handbook on the theory and methods of differential item functioning (DIF): logistic regression modeling as a unitary framework for binary and likert-type (ordinal) item scores.* Ottawa ON: Directorate of Human Resources Research and Evaluation, Department of National Defense; 1999.

2. Teresi JA: **Different approaches to differential item functioning in health applications. Advantages, disadvantages and some neglected topics.** *Med Care* 2006, **44(Suppl 3):** S152-S170.

3. Petersen MA, Groenvold M, Bjorner JB, Aaronson N, Conroy T, Cull A *et al*.: **Use of differential item functioning analysis to assess the equivalence of translations of a questionnaire.** *Qual Life Res* 2003, **12:** 373-385.
